# Single, community-based blood glucose readings may be a viable alternative for community surveillance of HbA_1c_ and poor glycaemic control in people with known diabetes in resource-poor settings

**DOI:** 10.3402/gha.v9.31691

**Published:** 2016-08-09

**Authors:** Daniel D. Reidpath, Nowrozy K. Jahan, Devi Mohan, Pascale Allotey

**Affiliations:** 1 Jeffrey Cheah School of Medicine and Health Sciences, Monash University Malaysia, Selangor, Malaysia; 2 South East Asia Community Observatory, Monash University Malaysia, Selangor, Malaysia

**Keywords:** diabetes, blood glucose, HbA_1c_, glycaemic control, community surveillance, developing countries

## Abstract

**Background:**

The term *HbA_1c_* (glycated haemoglobin) is commonly used in relation to diabetes mellitus. The measure gives an indication of the average blood sugar levels over a period of weeks or months prior to testing. For most low- and middle-income countries HbA_1c_ measurement in community surveillance is prohibitively expensive. A question arises about the possibility of using a single blood glucose measure for estimating HbA_1c_ and therefore identifying poor glycaemic control in resource-poor settings.

**Design:**

Using data from the 2011–2012 US National Health and Nutrition Examination Surveys, we examined the relationship between HbA_1c_ and a single fasting measure of blood glucose in a non-clinical population of people with known diabetes (*n*=333). A linear equation for estimating HbA_1c_ from blood glucose was developed. Appropriate blood glucose cut-off values were set for poor glycaemic control (HbA_1c_≥69.4 mmol/mol).

**Results:**

The HbA_1c_ and blood glucose measures were well correlated (*r*=0.7). Three blood glucose cut-off values were considered for classifying poor glycaemic control: 8.0, 8.9, and 11.4 mmol/L. A blood glucose of 11.4 had a specificity of 1, but poor sensitivity (0.37); 8.9 had high specificity (0.94) and moderate sensitivity (0.7); 8.0 was associated with good specificity (0.81) and sensitivity (0.75).

**Conclusions:**

Where HbA_1c_ measurement is too expensive for community surveillance, a single blood glucose measure may be a reasonable alternative. Generalising the specific results from these US data to low resource settings may not be appropriate, but the general approach is worthy of further investigation.

## Introduction

*Glycaemic control* is a medical term that refers to levels of sugar or glucose in the blood, with an emphasis placed on average blood glucose levels. Poor glycaemic control in people with known diabetes increases the risk of all-cause mortality and morbidity (1), including complications from cardiovascular disease (2, 3), kidney disease (4, 5), and eye disease (6, 7). Accurate testing for glycaemic control is important for diabetes research. In low- and middle-income countries (LMICs), community surveillance for glycaemic control is challenging because of the costs of blood testing.

Glycated haemoglobin (HbA_1c_) is routinely used as an indicator of average glycaemic control (8). The rate of haemoglobin (Hb) glycation – the bonding of a glucose molecule to an Hb molecule – is a function of plasma glucose concentration, with higher plasma glucose levels associated with higher rates of Hb glycation (8, 9). Once glycation has occurred, it is not reversed for the life of the glycated cell, and red blood cells have a life of about 2–3 months. As a consequence of the glycation process and the known average life of a red blood cell, measuring HbA_1c_ levels may be used as an estimate of average blood glucose levels over a period of 2–3 months prior to testing (8).

The HbA_1c_ test has proved to be an effective tool for monitoring glycaemic control. A quick search of PubMed revealed its widespread use in both clinical and community-based research. A PubMed search using the terms ‘hba1c and glycaemic control’ had 3,113 hits. Unfortunately, in LMICs HbA_1c_ tests remain too expensive for general use (10, 11). For example, in Malaysia, an upper-middle-income country, an HbA_1c_ test is 70 times more expensive than an equivalent blood glucose test. This is the reason why, in their national health surveys, countries like Malaysia and Thailand measure blood glucose at a single point in time rather than through an HbA_1c_ test, even in people with diagnosed diabetes (12).

When HbA_1c_ testing is not used in population surveys, the survey results are limited to estimating the prevalence of undiagnosed diabetes and the identification of risk factors (12–14). In the USA, the National Health and Nutrition Examination Surveys (NHANES) record measures of both blood glucose and HbA_1c_. Having both of these measures included in population-based research makes it possible to conduct studies of glycaemic control among people with known diabetes (15).

In the absence of adequate resources, the question arises: can a point-in-time blood glucose measure taken from people in the community known to have diabetes be used (1) to estimate HbA_1c_ and (2) to estimate glycaemic control?

Most research looking at HbA_1c_ and blood glucose has focussed on the relationship between HbA_1c_ and *average* blood glucose, as well as the estimation of average blood glucose from HbA_1c_ (16, 17). In one study, for example, investigators averaged seven readings (16), and in another investigators used eight readings over 1 day (17). A smaller but significant body of work has developed around the relationship between HbA_1c_ and a single blood glucose measurement – which can be either a fasting blood glucose measure or a random blood glucose measure (10, 11, 18–21). With one exception, the studies were motivated by the question posed here (19). Unfortunately, in at least two of the studies, the researchers confused the estimation of HbA_1c_ from blood glucose levels with the estimation of blood glucose levels from HbA_1c_ (22). Furthermore, all studies that looked at the relationship between HbA_1c_ and blood glucose were conducted in clinical settings rather than in the general community. In these clinical studies, subjects attended healthcare facilities as part of their routine care (18, 20). It is well known that the social, economic, and clinical profile of people with an illness who attend healthcare facilities is quite different from the profile of people who do not attend clinics (23–25). It is unclear whether the relationship observed between HbA_1c_ and blood glucose in clinical research is relevant for community surveillance. Nevertheless community surveillance is particularly pertinent in resource-poor settings.

Ideally, in LMICs the relationship between HbA_1c_ and blood glucose would be examined using data from a sample of community-dwelling people with diabetes. Unfortunately such data are not available. In their absence we examined the relationship using the best available data. In this study US population survey data from individuals who self-reported diabetes were used to develop a linear model to estimate HbA_1c_ (and therefore glycaemic control) from a single blood glucose measurement. A logistic model was also developed to classify people with diabetes as having good or poor glycaemic control based on that single blood glucose measurement. The results provide some insight into the potential utility of adapting this approach for low resource settings.

## Methods

NHANES 2011–2012 public use data sets were used for the study. NHANES is a multistage stratified, clustered probability sample of the civilian non-institutionalised population of the USA (26). This research using NHANES was approved by the Ethics Review Board of the US National Center for Health Statistics.

### Study population

**Fig. 1:**
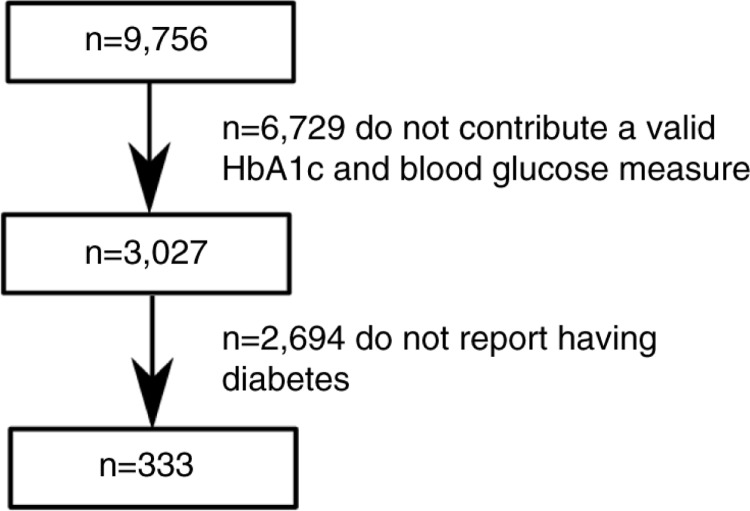
Sample selection flow diagram. Selection of the final sample of people with diabetes who contributed a valid blood sample from the US National Health and Nutrition Examination Surveys 2011–2012 public use data.

The total sample of NHANES in 2011–2012 was 9,756. In this study, participants were included only if they were aged 12 years and older, assessed in the morning examination session, contributed a valid blood sample for the measurement of blood glucose and HbA_1c_, and self-reported diabetes (Fig. 1). Self-reporting was based on a ‘yes’ response to the interview question, ‘other than during pregnancy, have you ever been told by a doctor or health professional that you have diabetes or sugar diabetes?’ Valid blood glucose and HbA_1c_ tests were contributed by 3,027 individuals, of whom only 333 self-reported diabetes: 175 males and 158 females. After the application of the exclusion criteria, the age range of participants decreased to 16–80 years. The median age of the sample was 63; the mean age was 61.7 years (SD=13.8).

### Data

Details of the data collection and coding conducted prior to the release of the public use data sets can be found on the Centers for Disease Control and Prevention website (wwwn.cdc.gov/nchs/nhanes/search/nhanes11_12.aspx). The target population for NHANES is the non-institutionalised, civilian, resident population of the USA. Each year approximately 5,000 individuals are interviewed in their homes and complete a health examination in mobile examination clinics.

Blood glucose values were based on fasting blood glucose samples analysed using an enzymatic assay conducted at the Fairview Medical Center Laboratory at the University of Minnesota. Data were originally reported in mg/dL and were converted to mmol/L prior to release of the public use data set.

HbA_1c_ measurement was performed on blood samples using a Tosoh Medics A_1c_ G7 HPLC Glycohemoglobin Analyzer. Results were reported in percentage units in the public use dataset and, following the International Federation of Clinical Chemistry standard, were converted to millimoles per mole (mmol/mol) for the statistical analyses reported here (27).

Poor glycaemic control has no fixed HbA_1c_-based criterion. Researchers have found elevated risk at various HbA_1c_ values or used various values in studies of glycaemic control (2, 28, 29). In this study poorly controlled diabetes was operationalised using a cut-off of 69.4 mmol/mol (8.5%) based on results from a study showing an increased risk of cardiovascular disease-related hospitalisation and all-cause mortality (3). HbA_1c_≥69.4 mmol/mol was coded 1 and otherwise 0.

### Statistical analysis

All analyses were conducted in the R statistical environment (30). A bivariable, linear regression model was developed to estimate HbA_1c_ from a single blood glucose measure. A survey-weighted estimation procedure was used that applied an iteratively weighted least squares algorithm (31). The complex survey methodology of NHANES was managed using the R ‘survey’ package, taking advantage of the sampling design (clusters, strata, and design weights) reported in the public use data sets (32). The design weights used in the analyses were for the fasting plasma blood glucose sub-sample in the full NHANES data set.

The linear equation was used to identify the blood glucose level associated with an HbA_1c_ of 69.4 mmol/mol and to create a classification table of actual and predicted poorly controlled diabetes. Sensitivity, specificity, and accuracy were calculated.

To extend the prediction model, an equivalent bivariable, logistic regression model was developed using an HbA_1c_ cut-off value of 69.4 mmol/mol to dichotomise poor glycaemic control. The sensitivity, specificity, and accuracy of various potential, predictive blood glucose values were subsequently examined.

In all cases, the classification tables used the weighted data, normalised to maintain the sample size of 333. Weighting the data in this manner produces correct design-based estimates of sensitivity, specificity, and accuracy, but the standard errors are likely to be incorrect and are not reported.

## Results

The mean population HbA_1c_ was estimated to be 58.97 mmol/mol (SE=1.00) with a lower median value (53.00 mmol/mol). This is indicative of a skewed distribution with a longer right tail. The interquartile range was wide (44.26–69.37), suggesting substantial variation in glycaemic control. The mean blood glucose was 8.77 mmol/L (SE=0.30) with a lower median value (7.71 mmol/L). This is also indicative of a skewed distribution. The interquartile range was 6.44–10.05 mmol/L.

There was a moderate population correlation between HbA_1c_ values and blood glucose values (*r*=0.7, *p*<0.0001). The equation to estimate HbA_1c_ ($\hat{y}$) from blood glucose (*x*) with the best least squares fit was:

1 $$\hat{y}=3.99x+24.01$$

**Fig. 2:**
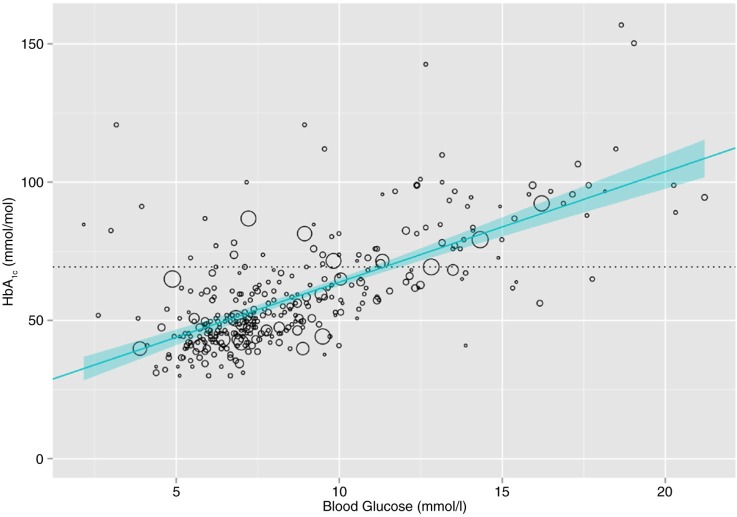
Estimation of HbA1c from blood glucose for a population of people with known diabetes using US National Health and Nutrition Examination Surveys 2011–2012 data.

The estimated slope and intercept were both significant (*p*<0.0001). Higher order terms for blood glucose were tested, did not add appreciably to the fit, and were subsequently excluded. The plot of the weighted linear bivariate relationship between the blood glucose and HbA_1c_ is shown in Fig. 2. The line of best fit and the 95% confidence intervals around the line are also shown. A horizontal, dashed line shows the HbA_1c_ values above which a person with diabetes would be classified as having poor glycaemic control, assuming a cut-off of 69.4 mmol/mol.

Applying Equation 1, a blood glucose of 11.4 mmol/L predicts an HbA_1c_ of 69.4 mmol/mol. This is the point at which the horizontal and regression lines in Fig. 2 intersect. Using these values to dichotomise the weighted sample into those with good and poor glycaemic control (actual from HbA_1c_ and predicted from blood glucose), we calculated the sensitivity, specificity, and accuracy of the classification (Table 1).

**Table 1:** Comparison of three blood glucose levels (11.4, 8.0, and 8.9 mmol/L) for the classification of good or poor glycaemic control based on an actual HbA_1c_ cut-point of 69.4 mmol/mol

	Blood glucose 11.4 mmol/L		Blood glucose 8.0 mmol/L		Blood glucose 8.9 mmol/L	
			
	Predicted good	Predicted poor	Predicted good	Predicted poor	Predicted good	Predicted poor
Actual good	165	0	134	31	155	10
Actual poor	106	62	42	126	51	117
Sensitivity	0.369		0.750		0.696	
Specificity	1		0.812		0.939	
Accuracy	68.2%		78.0%		81.7%	

All 62 cases predicted to have poor glycaemic control (based on blood glucose) did actually have poor glycaemic control (based on HbA_1c_). There were no false positive cases. However, the sensitivity was low, only correctly identifying 36.9% of all those with poor glycaemic control.

Given that classification of poor glycaemic control was the second goal of the study, not just the prediction of HbA_1c_ values, a logistic model was developed to estimate the probability of poor glycaemic control given a particular blood glucose level: *Pr*(*y*=1∣*x*), where *y* is the classification of actual HbA_1c_ values, and *x* is the blood glucose values. The logistic model was as follows:

2 $$\hat{y}=\left(\frac{\hat{p}}{1-\hat{p}}\right)=0.457x-5.485$$

3 $$\hat{p}=\frac{e^{0.457x-5.485}}{1+e^{0.457x-5.485}}$$

**Fig. 3:**
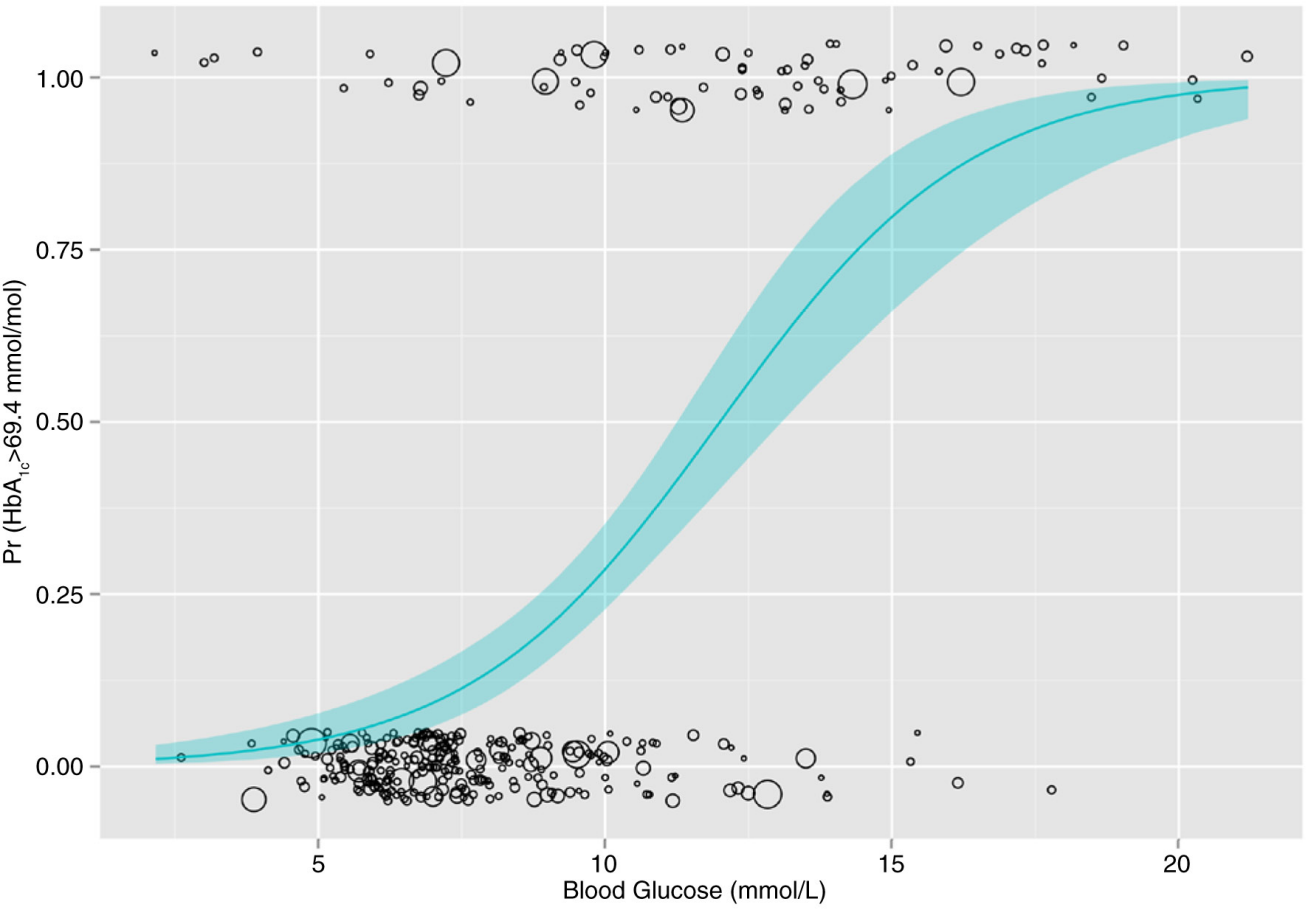
Estimation of poorly controlled diabetes predicted by blood glucose, based on an HbA_1c_ cut-off of 69.4 mmol/mol for a population with known diabetes using NHANES 2011–2012 data.

The slope and intercept of the parameters in Equation 2 were both significant (*p*<0.0001). The plot of the weighted, logistic, bivariate relationship between the blood glucose and HbA_1c_ is shown in Fig. 3 with the logistic curve and the 95% confidence intervals around the curve.

The wider dispersal of blood glucose values in people with known diabetes with poor glycaemic control (the points at the top of Fig. 3) compared with those with good glycaemic control (the points at the bottom) highlights the challenge of using a single blood glucose measure as a proxy for classification of glycaemic control. There is, unfortunately, no correct answer about the best blood glucose value for classifying poor glycaemic control, and it depends entirely on the purpose of the classification. Nonetheless, having examined the receiver operating characteristic curve (supplementary material) and the sensitivity, specificity, and accuracy for various blood glucose values, there appear to be two other candidate blood glucose values that may be more suitable for general community surveillance: 8.0 mmol/L and 8.9 mmol/L (Table 1).

A blood glucose of 8.9 mmol/L had the greatest accuracy among all possible blood glucose values for the classification of poor glycaemic control (81.7). The specificity was also very high (0.939) but with a concomitantly poorer sensitivity (0.696). A lower blood glucose value of 8.0 mmol/L had a slightly lower accuracy (78.0%) but arguably a better balance of sensitivity (0.750) against specificity (0.812).

## Discussion

In this study we examined the question of whether a single blood glucose measure could be used as a proxy for HbA_1c_ and its potential role in community surveillance of poor glycaemic control. The observed relationship between a single blood glucose value and HbA_1c_ was generally similar to previous *clinical* studies (18–20, 33). If the purpose is to estimate population HbA_1c_ or to look at the relationship between risk factors and a continuous measure of glycaemic control, then a single blood glucose measure could provide important insights into people living with diabetes in resource-poor communities.

Most LMICs will (or already do) face a significant chronic disease burden, including a burden from diabetes, and this burden is likely to increase into the foreseeable future (34). The strategy for managing large populations with diabetes will generally be devolved to government (Ministries of Health), and in LMICs management is likely to be implemented through primary healthcare or community healthcare facilities (35). The cost of routine HbA_1c_ surveillance will be prohibitively expensive for many governments. Inexpensive blood glucose measurement could provide a credible alternative for examining the impact of overall strategies, without necessarily providing any significant insight into individual patients. Using the lower blood glucose values of 8.0 or 8.9 mmol/L, for instance, it may be possible to provide some ‘policy sense’ about levels of glycaemic control within the community from a random sample of single blood glucose measures.

### Clinical implications

While this study was not about clinical management it would be remiss of us not to comment on the clinical utility of a single blood glucose measure. The reality appears to be that it is not ideal for identifying poor glycaemic control in a specific person with diabetes. In this study a high blood glucose (11.4 mmol/L) value identified with certainty a third of the people with poor glycaemic control; it missed two-thirds of them, and it had a zero false positive rate – high specificity, but low sensitivity. At least one clinical researcher, Mengesha (11), rejected blood glucose measures as a potential HbA_1c_ proxy for this very reason, citing its poor clinical value. Neither Mengesha nor Rosediani et al. (18), however, seemed to appreciate that by varying the blood glucose cut-off values one could adjust the sensitivity and specificity of the classification of glycaemic control for clinical purposes. However, it is beyond the scope of this study to consider it further.

### Ethics

The study does raise an important ethical question, which arises from the disjunctive value of blood glucose for clinical management versus community surveillance. There appears to be merit in using an imperfect (blood glucose) measure for the surveillance of glycaemic control in community-dwelling people with diabetes. When drawing a random sample of people with diabetes from the community, what obligation is there to refer a person with a specific blood glucose level for clinical evaluation/management? The higher the blood glucose cut-off for referral, the more certain we can be that the person has poor glycaemic control. We would not be wasting precious clinical resources on people who do not need them. On the other hand, the higher the cut-off, the more certain we can be that other people with poor glycaemic control in the sample will have been missed. What should the balance be between clinical management and population surveillance? We do not have an answer. This is an important policy question for each health system or research team according to its available resources. We flag the question here, however, to remind people of the potential ethical issues arising from chronic disease surveillance.

### Strengths

There are two important strengths of this study. First, the HbA_1c_ and blood glucose data come from a random sample of people known to have diabetes and living in the community, rather than from a clinical sample. This makes the study the first to have looked at the merits of blood glucose measurement for population surveillance of glycaemic control using an appropriate sample. The second strength of the study comes from the high quality of the NHANES methodology, which increases one's confidence about the accuracy of the blood glucose and HbA_1c_ measures, and the sampling strategy.

### Limitations

An important limitation of this study, however, is whether results from US community data can be generalised to lower income settings. Speculatively, we would imagine that the direction of the results are correct, but further basic measurement research should be undertaken in relevant resource-poor settings.

While a single blood glucose measure is not as good as HbA_1c_ for identifying poor glycaemic control, it is so much cheaper than HbA_1c_ that it warrants further investigation. Indeed, leveraging the widely accepted work of Nathan et al. (17) on the relationship between HbA_1c_ and *average* blood glucose, it may well be worth investigating the use of a few blood glucose tests taken over a week or two to estimate HbA_1c_.

## Conclusion

There is an increasing burden of diabetes in LMICs. Glycaemic control is central to the management of diabetes, but the standard measure of glycaemic control (HbA_1c_) is beyond the financial reach of Ministries of Health in most LMICs. This cost impediment is as true for clinical management as it is for research and community surveillance. A single blood glucose measure may be suitable for surveillance purposes and could provide important policy insights into the adequacy of diabetes care policies that are being implemented. Additional research would be required in resource-poor settings before firm recommendations could be made.

## Paper context

We were motivated by exactly the question the paper seeks to answer. Can a single blood glucose value be used to estimate glycaemic control in a known diabetic population? This is critical in settings where HbA_1c_ tests are too expensive for routine community surveillance. In the absence of appropriate data from LMICs, we use data from the US NHANES survey as a proof of principle. The indication is that single blood glucose measures are a promising alternative for surveillance in resource-poor settings.
